# A Pragmatic Oscillome: Aligning Visual Attentional Mechanisms with Language Comprehension

**DOI:** 10.3389/fnsys.2016.00072

**Published:** 2016-08-26

**Authors:** Elliot Murphy

**Affiliations:** Division of Psychology and Language Sciences, University College LondonLondon, UK

**Keywords:** neural oscillations, cross-frequency coupling, pragmatics, oscillome, relevance theory

A growing body of work over the last decade has investigated the potential functional role of neural oscillations in language comprehension (Giraud and Poeppel, 2012; Doelling and Poeppel, 2015; Lewis and Bastiaansen, 2015; Ding et al., 2016). I will explore how a number of recent developments in the field, and related domains of systems neuroscience, can generate much-needed linking hypotheses between the language sciences and neuroscience. To this end, I will focus on an area of linguistics whose existence has barely been acknowledged by the oscillation literature—pragmatics—and argue that elementary principles of discourse interpretation (though not more complex, peripheral aspects of pragmatic knowledge) can be implemented via generic, domain-general mechanisms elsewhere argued to be responsible for particular aspects of visual cognition. It will be suggested that these two systems share a number of striking computational/representational properties, and hence may share homologous dynamic and connectomic substrates.

Beginning first with the visual system, Jensen et al.'s (2012) approach to the prioritization of salient unattended stimuli claims that neocortical γ rhythms phase-lock to posterior α- and β-oscillating regions to form a clocking mechanism which activates sequences of visual representations. The striate cortex consequently extracts different features from distant regions, aiding the construction of a coherent visual scene. This process ensures that object X in a given scene is interpreted before object Y, imposing general and efficient set-constructing rules. These proposals are in accordance with the broader consensus that α phases modulate neuronal excitability and γ activity. This is a particular manifestation of what we could call “Wallace”s Problem' after David Foster Wallace: How does the brain deal with the sheer mass of sensory overload it receives constantly? [“What always amazed Wallace about real life was the overload of information,” writes his biographer Max (2013:p. 244)].

I will argue that if similar “oscillomic” (referring to a specific feature of brain dynamics, namely neural oscillations) mechanisms are responsible for the construction of linguistic feature-sets, then the principles of a particular theory of pragmatics, Relevance Theory, could be neurobiologically grounded. Relevance Theory claims that during discourse comprehension particular representations are triggered before others due to their “cognitive relevance” (Sperber and Wilson, 1995). The Communicative Principle of Relevance claims that “Every utterance (and ostensive stimulus more generally) coveys a presumption of its own optimal relevance,” relatedly, the Relevance-Theoretic Comprehension Procedure states that language comprehenders follow a path of least effort in computing cognitive effects (Wilson and Sperber, 2004). Processes involving lexical pragmatics (Wilson and Carston, 2007) adjust or modulate existing elements of linguistic meaning, as in the case where “David is a man” is interpreted as meaning David is an IDEAL MAN, with the lexical item *man* being underspecified for its ultimate meaning due to its ambiguity (Murphy, 2016a). From these basic processes we can already see a certain degree of similarity with visual attentional processes which construct representations based on factors such as salience, prominence and accessibility.

It was suggested in Murphy (2016b) that the neural ensembles responsible for storing representations used to construct linguistic phrases require certain phase-amplitude-locking levels in order for the regions coupled with them to “read off” their content. This would permit only certain features to be interpreted at the conceptual interfaces. Lisman and Jensen (2013) claim that coupled γ and θ oscillations form a code for representing multiple, sequenced items. These rhythms are generated in the cortex (in particular, occipital regions) and hippocampus. It may be, then, that the construction of linguistic feature-sets proceeds via the deployment of a similar oscillatory mechanism.

In the model of feature-set retrieval outlined in Figure 1, after inhibition reduces over the θ cycle the most excitable clusters would be itemized through a series of γ cycles. Less excitable representations would then follow, determining the make-up of a given feature-set. The group of feedforward γ rhythms required would be mostly generated in supragranular cortical layers (L2/3) (Maier et al., 2010) and hippocampal θ would be generated via slow pulses of GABAergic inhibition as a consequence of medial septum input, being part of a brainstem-diencephalo-septohippocampal θ-generating system (Vertes and Kocsis, 1997). The model in Figure 1 therefore permits the feeding of distributed conceptual and visual representations into hippocampal and posterior systems, binding the most excitable, cognitively relevant features (with this process doubtless involving a number of subcortical structures like the basal ganglia and thalamus). As a mechanistic basis generating a major component of Wallace's Problem, for pragmatic interpretation γ-θ coupling is required, whereas γ-α/β coupling is responsible for vision. Jensen et al.'s (2012) visual attention mechanism may also interface with a similar mechanism responsible for conceptual/pragmatic interpretation, such that in some cases the visually most prominent feature is also the most pragmatically relevant feature. Indeed, there may be some causal connection between the two: the more ancient visual attentional mechanism may determine pragmatic relevance in some cases, such that the prominence of certain visual features influences the interpretation of communicative referential intent. Consider the following two sentences in a context in which the objects under discussion are nearby (where “^*^” denotes an unacceptable interpretation and “?” denotes a less likely one):

(1) a. Your car is dirty [*the frame/the passenger cabin*/^*^the engine].
b. My computer is broken [*the processor/?the screen*].

**Figure 1:**
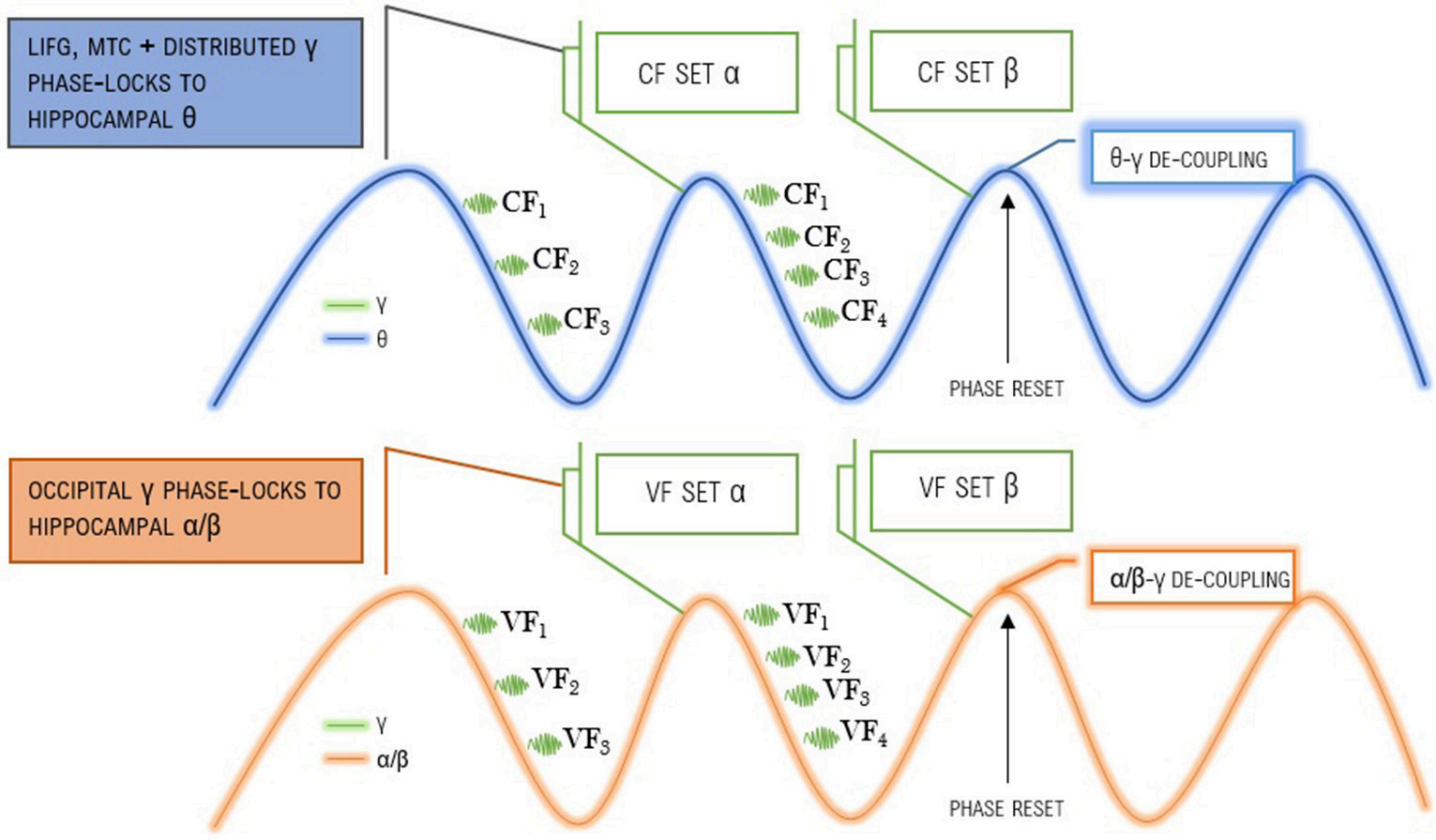
**A Relevance Theory-inspired oscillomic model of language comprehension**. “CF” denotes conceptual feature, “VF” denotes visual feature, “LIFG” denotes left inferior frontal gyrus, “MTC” denotes middle temporal cortex. The top image represents the proposed pragmatic oscillomic mechanism, and the bottom image refers to Jensen et al.'s (2012) model. See Murphy (2015, 2016b) for related discussion, and also Voloh and Womelsdorf (2016) for evidence that phase resetting to endogenous or exogenous cues facilitates information transfer between distributed brain areas, supporting its presently proposed role in feature-set composition (with such feature-sets being interpretable by conceptual systems typically seen as being widely distributed across the neocortex).

In (1a), visual attention mechanisms would phase-lock with pragmatic mechanisms via coupling and connectivity across a frontal-occipital-hippocampal network known to be responsible for visual object processing, and where transient β coupling between these three regions has been detected (Sehatpour et al., 2008), but in (1b) they would not be in phase. Cases like (1b) produce a more distant relation between visual and semantic representations. In (1a) two cognitive systems, visual attention and pragmatic interpretation, interface in some way to achieve the desired interpretation. This alignment is not found in (1b). Of course not all cases of salient stimuli would involve a direct alignment between visual and semantic representations, but the present claim is that those that do would be implemented via the above oscillomic processes of feature-set composition. The conceptual features required to construct the representation of a particular object or event would be combined via the above algorithm of fast γ rhythms being embedded within slower rhythms originating in language- and memory-related neural circuits.

The urge to maximize cognitive relevance may stem, then, from oscillomic processes homologous to those responsible for the visual system's urge to interpret particular features of a scene in a given order, with different representations becoming active at different stages of the slow θ*/*α cycle. What is deemed linguistically relevant would therefore be a matter not simply for external stimuli, but would rather be dependent on and constrained by internal brain events like the phase of particular oscillations. This therefore extends the schematic proposal in Murphy (2016b) to a more specific domain of linguistic interpretation. Pragmatic processes of optimizing relevance seem computationally suited to the ensemble activation operations produced via brain rhythm couplings. There is doubtless much more to pragmatics than simply activating the most cognitively relevant representations after processing a given utterance (for instance, I have not discussed the importance of ostensive-inferential communication), but the present proposal is meant only to explore the most essential, elementary features of pragmatic competence.

While phase-locked visual representations are generated by being presented to the eyes at the same time, linguistic information is necessarily processed in sequence, not during any given instant. But this still leaves considerable room for oscillatory dynamics to track previously processed visual information and attempt to match it with the input from a given word. The present claim is not that an entire sentence triggers the accessing of stored representations which are ultimately accessed as a function of their excitability; rather, it is that on the occasion of processing a given word (e.g., *chair*) these phase-locking operations would occur. Two distinct neural systems (one centered in occipital regions, the other in more left inferior frontal and hippocampal regions) would then implement symmetrical oscillatory processes to achieve similar goals, producing the most visually and linguistically relevant representations.

Finally, there are a number of experimental and theoretical possibilities which open up at this point. Future work could enrich the design of Jensen et al. (2012) to expose participants to a number of scenarios which modulate the alignment of pragmatic and visual relevance, while related electrocorticographic research could begin to track the dynamics of relevance-theoretic principles. Empirically testing the present model could involve the use of EEG and MEG, with scenarios of varying degrees of alignment between visual and linguistic relevance being presented to subjects, tracking the dynamics in brain regions hypothesized to be their neurocomputational loci.

## Author contributions

The author confirms being the sole contributor of this work and approved it for publication.

### Conflict of interest statement

The author declares that the research was conducted in the absence of any commercial or financial relationships that could be construed as a potential conflict of interest.
